# Targeting GRB7/ERK/FOXM1 Signaling Pathway Impairs Aggressiveness of Ovarian Cancer Cells

**DOI:** 10.1371/journal.pone.0052578

**Published:** 2012-12-20

**Authors:** David W. Chan, Winnie W. Y. Hui, Patty C. H. Cai, Michelle X. Liu, Mingo M. H. Yung, Celia S. L. Mak, Thomas H. Y. Leung, Karen K. L. Chan, Hextan Y. S. Ngan

**Affiliations:** Department of Obstetrics and Gynaecology, LKS Faculty of Medicine, The University of Hong Kong, Hong Kong SAR, P.R.China; Barts & The London School of Medicine and Dentistry, Queen Mary University of London, United Kingdom

## Abstract

Ovarian cancer is a highly lethal disease with poor prognosis and especially in high-grade tumor. Emerging evidence has reported that aberrant upregulation and activation of GRB7, ERK as well as FOXM1 are closely associated with aggresivenesss of human cancers. However, the interplay between these factors in the pathogenesis of human cancers still remains unclear. In this study, we found that GRB7 (*P*<0.0001), ERK phosphorylation (*P*<0.0001) and FOXM1 (*P* = 0.001) were frequently increased and associated with high-grade tumors, as well as a high tendency in association with advanced stage ovarian cancer by immunohistochemical analysis. Intriguingly, the expressions of GRB7 (*P*<0.0001), ERK phosphorylation (*P*<0.001) and FOXM1 (*P*<0.001) showed a significant stepwise increase pattern along Grade 1 to Grade 3 ovarian cancers. Biochemical studies using western blot analysis demonstrated that enforced expression or knockdown of GRB7 showed GRB7 could elevate the levels of ERK phosphorylation and FOXM1, whereas enforced expression of FOXM1 could not alter levels of GRB7 and ERK phosphorylation. But inhibition of ERK signaling by U0126 or PD98059 could reduce the level of FOXM1 in GRB7-overexpressing ovarian cancer cells, suggesting that GRB7, ERK and FOXM1 are regulated orderly. Moreover, inhibition of ERK activity by U0126 or PD98059, or decreased FOXM1 expression by Thiostrepton significantly inhibited cell migration/invasion, tumor growth *in vitro* and *in vivo*. Collectively, our findings confer that targeting GRB7/ERK/FOXM1 signaling cascade may be a promising molecular therapeutic choice in combating ovarian cancer.

## Introduction

Ovarian cancer is the most lethal disease among all gynecological malignancies [1], [2]. The high mortality rate of ovarian cancer is because of poor prognosis and most cases are detected at late stage. Ovarian cancer is a heterogeneous tumor exhibiting a wide range of cytological, clinical and altered genetic features [3], [4]. Ovarian high-grade cancers are characterized by high-grade nuclei, a high mitotic index, more aggressiveness, poorly differentiated, less responsive to chemotherapy as well as low survival rate [4], [5], [6]. The relatively worse pathogenesis and clinico-pathologic features of high-grade ovarian cancers cause poor clinical management of this type of disease. Therefore, understanding the underlying molecular mechanisms may assist in developing better curative therapy in aggressive ovarian cancers.

Growth factor receptor-bound protein 7 (GRB7) is a signaling adaptor which functions to couple signals from cell surface receptors to specific signaling pathways [7]. Emerging evidences have reported that GRB7 is overexpressed and usually accompanied with overexpression of erbB2 in human cancers [8], [9]. Clinicopathological analyses have shown that the upregulated GRB7 is associated with metastatic behavior [8], [10], [11], [12]. Besides, constitutive activation of ERK has been implicated in a variety of tumorigenic behaviors such as cell proliferation, differentiation, metastasis, angiogenesis, and chemoresistance in human cancers [13], [14], [15], [16]. In addition, Forkhead Box M1 (FOXM1) has been identified as an oncogenic transcription factor which is frequently upregulated in numerous human cancers [17], [18], [19]. We and others have recently found that the upregulation of FOXM1 enhances cell proliferation, migration/invasion and particularly its expression is involved in cancer progression [17], [20], [21], [22]. Interestingly, the above three factors seem to converge towards the aggressive cancer properties such as high-grade and advanced stage tumors, yet the relationship of their regulatory mechanism has not been completely elucidated. Therefore, the delineation of the interplay of their regulatory mechanisms will assist in exploring a potential therapeutic target in ovarian cancer.

In this study, we show that the expressions of GRB7, ERK and FOXM1 were significantly correlated with the progression of ovarian cancer. We also describe the regulatory mechanism of GRB7/ERK/FOXM1 signaling cascade and the inhibition of this signaling using either the chemical inhibitor U0126 or FOXM1 inhibitor Thiostrepton could remarkably abrogate the ovarian cancer cell migration/invasion, and *in vitro* and *in vivo* tumor growth. Our results emphasize the importance of the GRB7/ERK/FOXM1 pathway in tumorigenic properties and argue this pathway for a promising therapeutic target in high-grade ovarian cancer.

## Materials and Methods

### Cell Lines and Drugs

Two ovarian cancer cell lines: A2780cp (gifts from Prof. BK Tsang, Dept. of Obstetrics & Gynaecology, University of Ottawa) [23], and OVCA433 (obtained from American Type Culture Collection, Rockville, MD), as well as two GRB7 stably expressing clones; C19 in OVCA433 and C15 in A2780cp which were generated previously [12] were included in this study. All were grown at 37°C in 5% CO_2_ in minimum essential medium or Dulbecco’s modified Eagle medium supplemented with 10% fetal bovine serum. MAPK/ERK kinase 1/2 (MEK1/2) inhibitors PD98059 and U0126, and FOXM1 inhibitor Thiostrepton were obtained from Calbiochem (La Jolla, CA, USA).

### Plasmids and Cell Transfection

The pEGFP/GRB7 expressing plasmids were used as previously [12]. Four shRNA HuSH 29mer shRNA constructs against GRB7 in pGFP-V-RS vector were purchased from OriGene Technologies for generating stable GRB7 knockdown cells (Cat. No. TG312621, OriGene Technologies, Inc, Rockville, MD, USA). The non-effective 29-mer scrambled shRNA (TR30013) (OriGene Technologies) was used as a negative control. To knockdown human FOXM1, the TriFECTa® RNAi Kit which contains three siRNAs targeting human FOXM1 was purchased from IDT (Integrated DNA Technologies, Inc., Iowa, USA). Cell transfection was carried out using LipofectAMINE™ 2000 (Invitrogen) according to the manufacturer’s instructions. The expression patterns were analyzed by Western blotting. The parental vector pEGFP-C1 was used as empty vector control.

### Immunohistochemical and Western Blot Analyses

Immunohistochemical (IHC) staining for GRB7, ERK phosphorylation and FOXM1 was performed on an ovarian cancer tissue array (OVC1021) (Pantomics Inc, San Francisco, CA) using primary polyclonal anti-GRB7 (Santa Cruz Biotechnology, Inc., Santa Cruz, CA), anti-phospho-ERK (Chemicon International, Inc., Temecula, USA), and anti-FOXM1 (Abcam, Inc., Cambridge, MA, USA). The percentage of immuno-positive cells in tumors and normal epithelia was assessed by the proportions of immuno-positive cells ranged from 10 to 100%, and the intensity of staining scored as 0 (negative), 1 (faint), 2 (moderate), 3 (strong) and 4 (marked). The immunoreactivity for each case was scored as a percentage of the proportions of immuno-positive cells multiplied by the intensity of staining. The fold change of each staining was obtained by dividing the expression level of each cancer sample by the mean immunoreactive staining value of normal ovaries and borderline mixed cystadenoma. The quantification of immunohistochemical staining was scored blindly at least by two independent observers.

For Western blot analysis, cells were lysed with Cell Lysis Buffer (Cell Signaling Technology, Beverly, MA) containing Protease Inhibitor Cocktail (Roche, Indianapolis, IN) and PMSF (phenylmethylsulphonyl fluoride) (Sigma Chemical Co. St Louise, MO). The samples were resolved by SDS-PAGE and electroblotted onto Immobilon-P Transfer Membrane (Millipore Corporation, Bedford, MA). Blots were blocked with 5% skim milk, followed by incubation with anti-GRB7, FOXM1 (Santa Cruz), GFP (Abcam), phospho-ERK, ERK (Cell Signaling), and *β-*actin (Sigma Chemical Co., St Louis, MO). Blots were then incubated with goat anti-rabbit or anti-mouse secondary antibody conjugate with horseradish peroxidase (Amersham Pharmacia Biotech, Cleveland, OH) and visualized by enhanced chemiluminescence (ECL) (Amersham).

### Cell Viability Analysis

Cell viability was measured by Cell Proliferation Kit II (XTT) for 5 days according to the manufacturer’s instructions (Roche). The data were collected from at least three independent experiments.

### Cell Migration and Invasion Assays

To quantify the cell migratory and invasive capacities of ovarian cancer cells, the Transwell cell migration and cell invasion assay kits (Chemicon International, Inc., Temecula, CA) were used in this study according to the manufacturer’s instructions. In brief, 1.5×10^5^ cells were resuspended in serum free culture medium (Thiostrepton, PD98059 or U0126 was added when needed) and seeded on the upper chamber, whereas the full medium was placed in the lower chamber as chemo-attractant. The plate was incubated at 37°C and 5% CO_2_ for 12 hrs for cell migration assay, and 48 hrs for cell invasion assay allowing cells to pass through the pores in the membrane. The cell migrated and invaded rates were calculated after cell staining and counting by using at least three different fields for each transwell filter. The experiments were repeated thrice.

### *In Vivo* Tumorigenicity Assay

To examine the *in vivo* effects of U0126 and Thiostrepton on tumor development, 5×10^6^ A2780cp cells were inoculated s.c. into *BALB/c nu/nu* female mice of 3–4 weeks of age and in groups of five. The tumor formation in nude mice was monitored for every 3 days. 25 to 50 µmol/kg of U0126 or 200 to 300 µmol/kg Thiostrepton (Calbiochem, La Jolla, CA, USA) was administered i.p. once for every 3 days with total of 4 injections into five nude mice when every tumor size became ∼3 mm in diameter. As a control group, DMSO alone was administrated i.p. for the same time of treatment. The tumor sizes were measured using slide calipers and were calculated by the following formula: volume = (width) ^2^*length*π/6. The tumor growth curves were plotted from the mean volume±SEM of tumors from 5 mice. The side effects such as body weight changes were monitored closely. All the animal experiments were approved by the University of Hong Kong Committee on the Use of Live Animals in Teaching and Research (CULATR No.2560-11).

### Statistical Analysis

The clinical parameters were analyzed by SPSS 13.0 software (SPSS, Chicago, IL). Fisher’s exact test (for parametric data) and Mann-Whitney test (for non-parametric data) were used to compare the values between subgroups. The Student’s *t*-test was used to analyze the cell viability, migration/invasion and *in vivo* tumor growth results. A *p*-value was considered significant when less than 0.05.

## Results

### GRB7, ERK Phosphorylation and FOXM1 are Frequently Elevated in Ovarian Cancer

**Figure 1:**
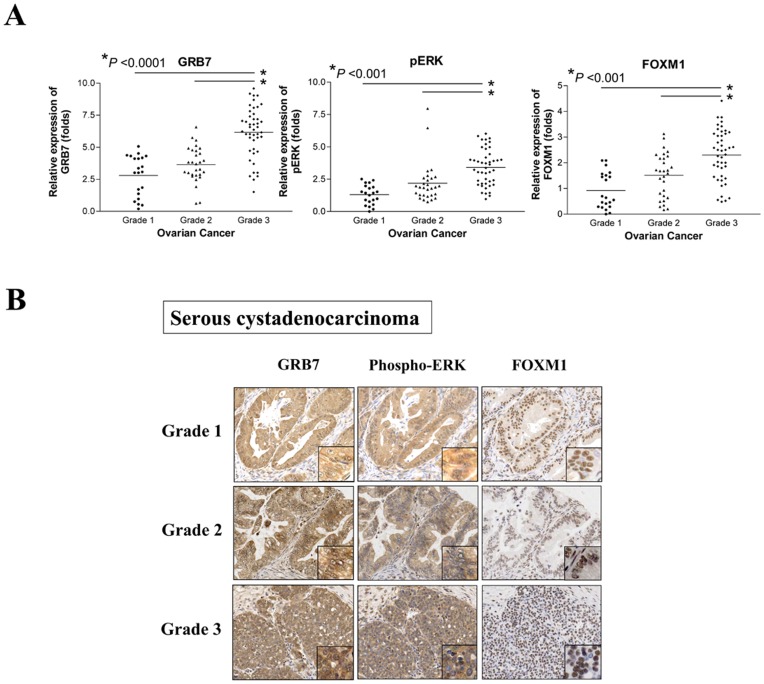
A significant stepwise increase in the expressions of GRB7, ERK phosphorylation and FOXM1 along ovarian tumor grade. (A) The expressions of GRB7, ERK phosphorylation and FOXM1 were evaluated by immunohistochemical analysis using specific antibodies on an ovarian cancer tissue array (OVC1021, Pantomics). (B) Representative pictures show the stepwise increase of GRB7, ERK phosphorylation and FOXM1 expressions from Grade 1 to Grade 3 ovarian cancer (serous subtype) (200× magnifications).

We have previously reported that GRB7 [12], ERK phosphorylation and FOXM1 [21] are overexpressed in ovarian cancer samples particularly in high-grade tumors. However, the interplay among these factors in ovarian cancers has not yet been elucidated. By immunohistochemical (IHC) analysis on an ovarian cancer tissue array (OVC1021), we found that all of these factors were congruently upregulated in ovarian cancer samples which was consistent with our previous findings [12], [21]. As expected, the overexpressed GRB7 (>4 folds) was correlated with the increased ERK phosphorylation (>2 folds) (*P*<0.0001, Fisher’s exact test) and FOXM1 (>3 folds) (*P*<0.0001, Fisher’s exact test) (Table 1). In addition, GRB7 (*P*<0.0001, Fisher’s Exact test), ERK phosphorylation (*P*<0.0001, Fisher’s exact test), and FOXM1 (*P* = 0.001, Fisher’s exact test) were significantly correlated with high-grade tumor and had a high tendency in association with advanced stage ovarian cancer (GRB7, *P* = 0.021; phospho-ERK, *P* = 0.065; and FOXM1, *P* = 0.065, Fisher’s exact test) (Table 1). It was noteworthy that a significant progressive increase of GRB7 (*P*<0.001, Mann-Whitney’s test), ERK phosphorylation (*P*<0.001, Mann-Whitney’s test), and FOXM1 (*P*<0.001, Mann-Whitney’s test) expression pattern was observed from Grade 1 to Grade 3 tumors (Fig. 1A and 1B) and a less obvious pattern from early to late stage ovarian cancers (Figure S1), suggesting that these factors play important roles in ovarian cancer progression. Taken together, these data indicate there is a close interplay among GRB7, ERK activity and FOXM1 in regulating ovarian cancer oncogenesis particularly in high-grade tumor.

**Table 1:** Clinico-pathological analysis of GRB7, ERK phosphorylation and FOXM1 expressions in ovarian cancer tissue array (OVC1021).

A				
	GRB7			
Parameters	n( = 96)	≤4-fold	>4-fold	*p*
*phospho-ERK*				
≤4-fold	42	30 (71%)	12 (29%)	
>4-fold	54	6 (11%)	48 (89%)	<0.0001*
*FOXM1*				
≤2-fold	58	31 (53%)	27 (47%)	
>2-fold	38	5 (13%)	33 (87%)	<0.0001*
**B**				
	**GRB7**			
**Parameters**	**n( = 96)**	**≤4-fold**	**>4-fold**	***p***
*Grade*				
Low	50	31 (62%)	19 (38%)	
High	45	4 (9%)	41 (91%)	<0.0001*
*Stage*				
Early	73	29 (40%)	44 (60%)	
Late	23	7 (30%)	16 (70%)	0.469
*Metastasis*				
Yes	23	7 (30%)	16 (70%)	
No	73	29 (40%)	44 (60%)	0.469
**C**				
	**phospho-ERK**			
**Parameters**	**n( = 96)**	**≤2-fold**	**>2-fold**	***p***
*Grade*				
Low	50	34 (68%)	16 (32%)	
High	45	7 (16%)	38 (84%)	<0.0001*
*Stage*				
Early	48	22 (46%)	26 (54%)	
Late	48	20 (42%)	28 (58%)	0.837
*Metastasis*				
Yes	23	9 (39%)	14 (61%)	
No	73	33 (45%)	40 (55%)	0.639
**D**				
	**FOXM1**			
**Parameters**	**n( = 96)**	**≤2-fold**	**>2-fold**	***p***
*Grade*				
Low	50	39 (78%)	11 (22%)	
High	45	19 (42%)	26 (58%)	0.001*
*Stage*				
Early	73	45 (62%)	28 (38%)	
Late	23	13 (57%)	10 (43%)	0.807
*Metastasis*				
Yes	23	13 (57%)	10 (43%)	
No	73	45 (62%)	28 (38%)	0.807

### Molecular Regulation of GRB7/ERK/FOXM1 Signaling in Ovarian Cancer Cells

**Figure 2:**
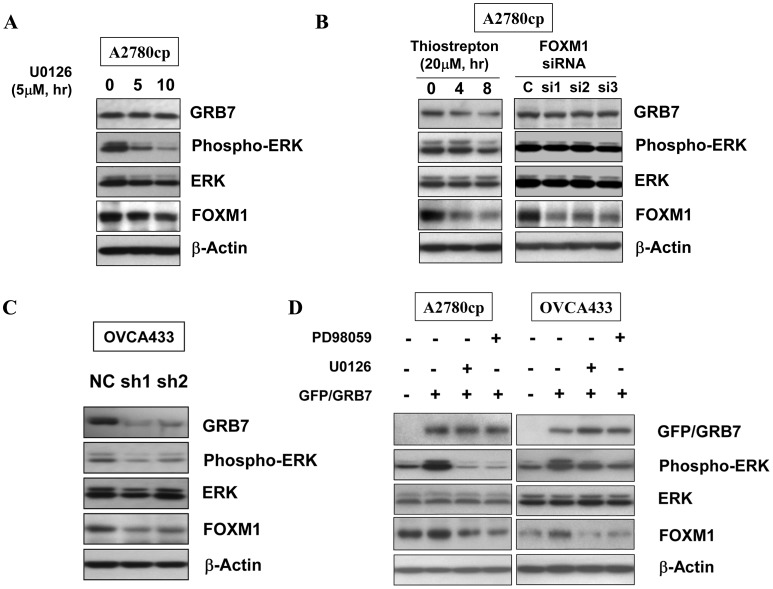
GRB7/ERK/FOXM1 was regulated in the same signaling axis. (A) Treatment with U0126 (5 µM) showed a significant reduction in the expression of ERK phosphorylation accompanied with FOXM1 in ovarian cancer cells time dependently, whereas no change in GRB7 expression was found in A2780cp cells. (B) Treatment of Thiostrepton (20 µM) remarkably reduced the expression of FOXM1 only but no change in the expressions of GRB7 and ERK phosphorylation in A2780cp cells (*left*). Depletion of FOXM1 by siRNA knockdown did not alter the expression of GRB7 and ERK phosphorylation (*right*). C, siRNA scrambled control. si1, si2 and si3 siRNAs targeting three different regions of human FOXM1 and knockdown the expression of FOXM1 by 60%, 45% and 70% respectively. (C) Two out of four GRB7 shRNA constructs (sh1 and sh2) showed ∼70% knockdown of GRB7 accompanied with a reduction of ERK phosphorylation and FOXM1 expressions in OVCA433 cells. The scrambled control (NC) was used as negative control. (D) Enforced expression of GRB7 increased ERK phosphorylation and FOXM1. However, treatment with either U0126 (10 µM) or PD98059 (20 µM) could suppress the induced ERK phosphorylation and FOXM1 in A2780cp and OVCA433 ovarian cancer cells.

Given that the increased GRB7, ERK phosphorylation and FOXM1 significantly correlate with high-grade ovarian tumor, they may be regulated coordinately in order to mediate oncogenic functions in ovarian cancer progression. We were thus interested to investigate the regulatory mechanism amongst these factors in ovarian cancer cells. For this purpose, both A2780cp and OVCA433 cells were firstly treated with U0126 MEK1/2 inhibitor. Western blotting showed that ERK phosphorylation and FOXM1 were remarkably reduced, but no change in GRB7 expression was observed (Fig. 2A). Besides, treatment of Thiostrepton successfully reduced the level of FOXM1, while the levels of GRB7 and ERK phosphorylation were still unchanged (Fig. 2B). To exclude the non-specific function of Thiostrepton in high-dose treatment, siRNA approach mediated FOXM1 knockdown was performed in A2780cp cells. Similar to the treatment of Thiostrepton, depletion of FOXM1 did not alter the expression of GRB7 and ERK phosphorylation (Fig. 2B). This indicates that the suppression of ERK activity could decrease FOXM1 level, whereas the reduction of FOXM1 expression does not affect either GRB7 expression or ERK activity. To further prove this observation, stably knockdown of endogenous GRB7 in a GRB7 high expressing cell line, OVCA433, using shRNA approach clearly showed that not only GRB7 but also ERK phosphorylation and FOXM1 were reduced (Fig. 2C). In contrast, enforced expression of GRB7 increased ERK phosphorylation and FOXM1 in A2780cp and OVCA433 cells (Fig. 2D). On the other hand, treatment of either U0126 or PD98059 MEK1/2 inhibitors could reduce not only ERK phosphorylation but also FOXM1 in GRB7 ectopic expressing cells (Fig. 2D). Collectively, these findings confer that GRB7 positively upregulates ERK activity which in turn elevates FOXM1 in ovarian cancer cells.

### Suppression of ERK Activity or FOXM1 Expression Decreases Ovarian Cancer Cell Migration and Invasion

**Figure 3:**
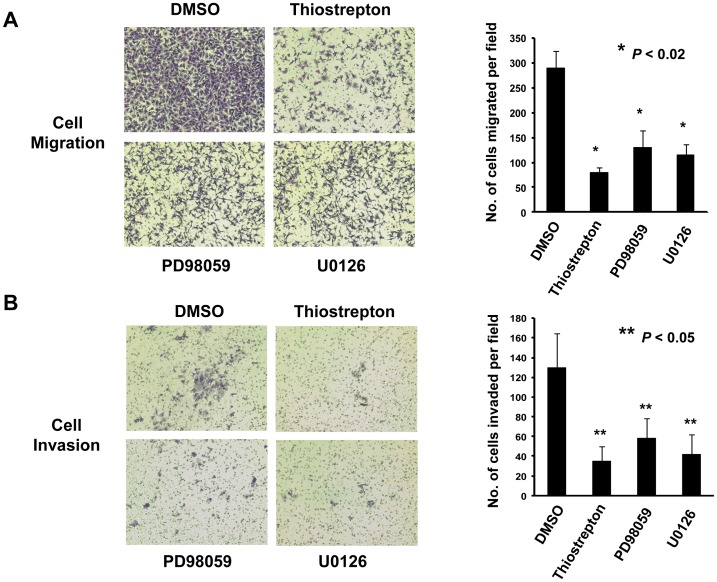
Inhibition of ERK phosphorylation or FOXM1 significantly decreased both migration and invasion of GRB7-overexpressing ovarian cancer cells. OVCA433 cells with stable expression of GFP/GRB7 (OVCA433-GRB7) were treated with DMSO as control, Thiostrepton (20 µM), PD98059 (20 µM) and U0126 (10 µM) for 6 hours and were analyzed by (A) Transwell cell migration assay. The representative pictures and bar chart showed significant reduction in the number of migratory cells through Matrigel-coated membrane in OVCA433-GRB7 cells treated with Thiostrepton, PD98059 and U0126 than DMSO control (*P<0.02, Student *t*-test) at 8-hour; (B) Transwell cell invasion assay. The representative pictures and bar chart showed significant reduction in the invasion rate in OVCA433-GRB7 cells treated with Thiostrepton, PD98059 and U0126 when compared with DMSO control (*P<0.05, Student *t*-test) at 15-hour.

It has been documented that high-grade ovarian tumor exhibits high capacity in cell migration/invasion [4], [24]. Thus, we wondered whether inhibition of ERK activity or FOXM1 expression by their specific inhibitors could influence the cell migration and invasion of ovarian cancer cells. To verify this hypothesis, a high-grade ovarian cancer cell line, OVCA433, displaying high migratory and invasive abilities was ectopically expressed with GFP/GRB7 and treated with inhibitors. Results showed that treatment of Thiostrepton, PD98059 and U0126 remarkably reduced cell migration rate of OVCA433-GRB7 cells by 3.5-fold, 2.2-fold and 2.5-fold respectively when compared with the control (Fig. 3A). Likewise, treatment of Thiostrepton, PD98059 and U0126 also significantly reduced cell invasion rate of OVCA433-GRB7 cells by 3.5-fold, 2.6-fold and 2.9-fold respectively as compared with the control (Fig. 3B). These findings suggest that the inhibition of GRB7/ERK/FOXM1 signaling is capable of impairing the cell migration and invasion of ovarian cancer cells.

### Targeting GRB7/ERK/FOXM1 Inhibits Ovarian Cancer Tumor Growth *in vitro* and *in vivo*

**Figure 4:**
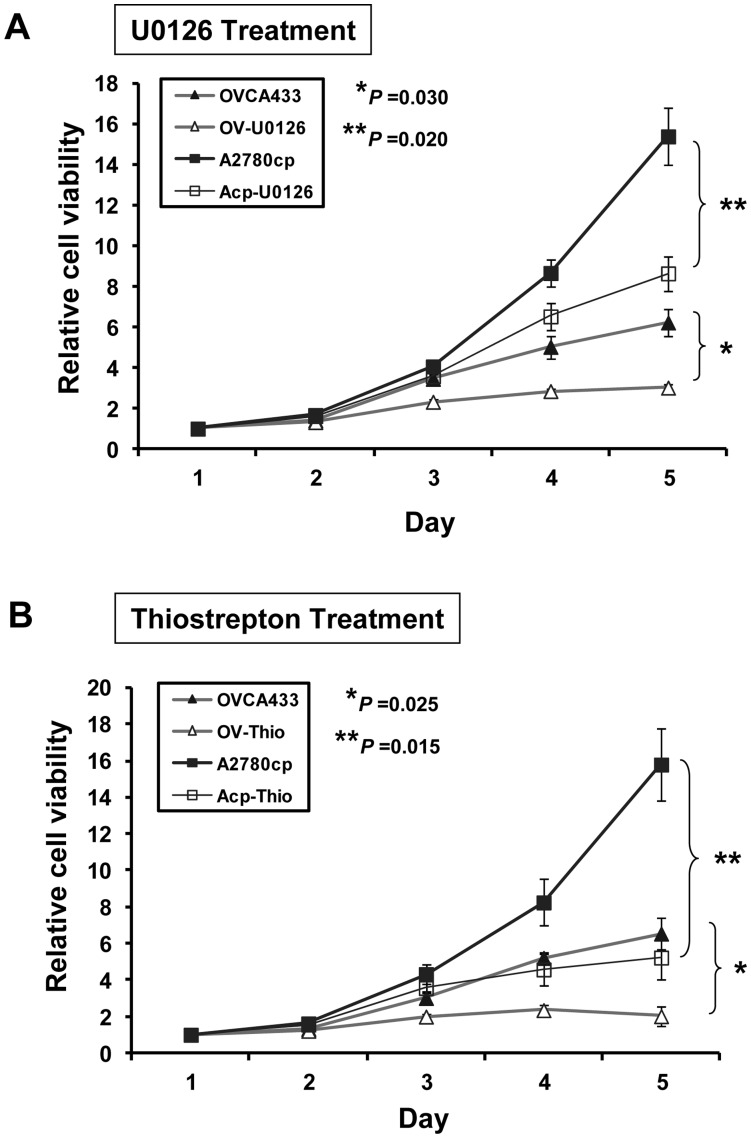
Inhibition of either ERK phosphorylation or FOXM1 expression impaired cell growth in ovarian cancer cells. (A) XTT cell proliferation assays showed that the inhibition of ERK phosphorylation by U0126 (10 µM) significantly abrogated the cell proliferation rate in GRB7 stably expressing OVCA433 cells (*P* = 0.020, Student *t*-test) and A2780cp cells (*P* = 0.030, Student *t*-test) as compared with their vector controls. (B) XTT cell proliferation assays showed that the suppression of FOXM1 expression by Thiostrepton (20 µM) significantly reduced the cell proliferation rate in GRB7 stably expressing OVCA433 cells (*P* = 0.015, Student *t*-test) and A2780cp cells (*P* = 0.025, Student *t*-test) as compared with their vector controls.

Previous studies have shown that constitutive activation of ERK activity or increased expression of GRB7 and FOXM1 are closely associated with tumorigenicity of various human cancers [12], [16], [21], [25], [26]. This suggests that targeting any components in this signaling cascade should theoretically abrogate the tumorigenic properties in ovarian cancer cells. Indeed, previous publications reported that knockdown of FOXM1 and ERK could reduce cell growth and invasiveness of human cancers [27], [28], [29]. In this study, we aimed to investigate the tumorigenic alterations by using pharmaceutical inhibitors specifically targeting GRB7/ERK/FOXM1 signaling cascade. We thus firstly evaluated the suppressive effect on cell proliferation of two high-grade serous ovarian cancer cell lines; A2780cp and OVCA433 using U0126 (10 µM). By XTT cell proliferation assay, both A2780cp (*P* = 0.02, Student *t*-test) and OVCA433 (*P* = 0.03, Student *t*-test) exhibited significant reduction in cell proliferation rate as compared with their controls (Fig. 4A). Similarly, upon treatment of Thiostrepton (20 µM), A2780cp (*P* = 0.015, Student *t*-test) and OVCA433 (*P* = 0.025, Student *t*-test) also showed a profound reduction in cell proliferation rate as compared with their controls (Fig. 4B).

**Figure 5:**
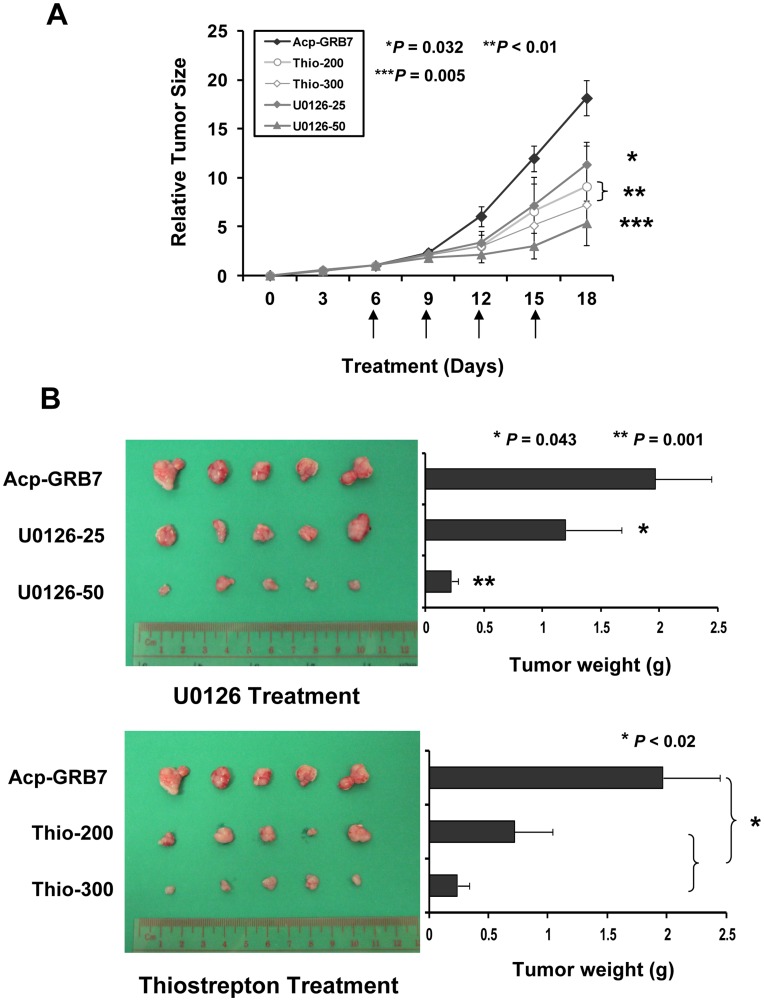
Inhibition of ERK phosphorylation or FOXM1 expression reduced tumor growth in a mouse xenograft model. (A) The GRB7 stably expressing A2780cp cells (Acp-GRB7) were subcutaneously injected into the right flank of nude mice. Mice were divided into 5 groups (5 mice per group) and treated with either DMSO as a control, or U0126 (25 or 50 µM/kg) or Thiostrepton (200 or 300 µM/kg) for every 3-day since on day 6 (Arrows represent the injections). The relative tumor size was calculated relative to those of the first day of treatment (day 0) and are represented as relative mean size (%)±SE for each group (*P = 0. 032, **P<0.01, and ***P = 0. 005, are significantly different from the DMSO control group, Student *t*-test). (B) The representative pictures and bar charts show the average tumor weight of each group taken on day 18. (*P = 0. 043, **P = 0. 001, and ***P<0.02, are significantly different from the DMSO control group, Student *t*-test).

We next examined the *in vivo* tumorigenic activity of GRB7 in A2780cp by subcutaneously inoculation GRB7 and vector control expressing A2780cp cells into one flank of nude mice. As expected, GRB7 stably expressing A2780cp cells exhibited 30% faster tumor growth as compared with the vector control (*P* = 0.020, Student *t*-test) (Figure S2). We next investigated the suppressive effect on tumor growth of GRB7 stably expressing A2780cp upon treatment of U0126 or Thiostrepton. When the tumor size reached ∼3 mm in diameter in tumor bearing nude mice on Day 6, U0126 at 25 and 50 µM/kg was i.p. injected into peritoneal cavity of nude mice for every 3-day. After 4 times of U0126 injection, we found that there were 35% and 72% reductions in tumor size as compared with DMSO control on Day 18 when injected with U0126 at 25 µM/kg (*P* = 0.032, Student *t*-test) and 50 µM/kg (*P* = 0.005, Student *t*-test) respectively (Figure 5A and 5B). Besides, upon treatment of Thiostrepton for 200 µM/kg and 300 µM/kg on Day 9, there were 47% and 52% reduction in tumor growth as compared with DMSO control on Day 18 respectively (*P*<0.01, Student *t*-test) (Figure 5A and 5B). These findings highlight that the upregulation of GRB7 could enhance tumor growth, while using either U0126 or Thiostrepton could efficiently reduce the tumor growth of ovarian cancer cells both *in vitro* and *in vivo*.

## Discussion

In this study, we present evidence that GRB7, ERK phosphorylation and FOXM1 are increased in ovarian cancer. Interestingly, the concomitant increase of GRB7, ERK phosphorylation and FOXM1 is associated with high-grade ovarian cancers. Importantly, we show that these factors are coordinately regulated in GRB7/ERK/FOXM1 signaling axis in ovarian cancer cells. Functionally, inhibiting ERK activity by U0126 or PD98059 and FOXM1 expression by Thiostrepton remarkably inhibit ovarian cancer cell migration/invasion and tumor growth *in vitro* and *in vivo*. These findings clearly evidence that the activated GRB7/ERK/FOXM1 signaling cascade plays an important role in the pathogenesis of high-grade ovarian cancer.

Constitutive activation of ERK signaling pathway has been involved in human cancer development [30], [31], [32]. Indeed, targeting this pathway to combat human cancers has been attracted plenty of effort to develop effective drugs for the past decade [15], [33], [34], [35]. We and others have recently found that GRB7 and its variant, GRB7v, are frequently upregulated in ovarian cancers and are able to enhance cell proliferation, migration/invasion through activating ERK signaling pathway [12], [36], [37]. On the other aspect, emerging evidences have found that the aberrant upregulation of FOXM1 transcription factor is involved in the pathogenesis of various human cancers [17], [21], [22], [38], [39]. Although all of these aberrantly activated factors are dominantly associated with cancer pathogenesis, there is no report so far to mention the signaling link amongst GRB7, ERK activity and FOXM1 in human cancer cells. In this study, by using IHC analysis of ovarian cancer tissue array, we show that GRB7, ERK phosphorylation and FOXM1 are concomitantly increased in ovarian cancer samples. Intriguingly, their expressions exhibit a stepwise increase along the tumor grade of ovarian cancers. In fact, our clinicopathological correlation analysis provides further evidence that their upregulated expressions are significantly associated with high-grade tumor. As we know, this is the first report showing the expression status of these factors involved in the progression of ovarian cancers thus far. All in all, these findings indicate that GRB7, ERK phosphorylation and FOXM1 form an oncogenic convergence in high-grade ovarian cancer pathogenesis. Therefore, targeting this signaling cascade may achieve a good outcome when treating this type of disease.

GRB7 is a known adaptor which relays signals from cell surface receptors to specific downstream signaling cascades via the protein-protein interaction of its Src-homology 2 (SH2) domain to a variety of tyrosine kinases [7], [40], [41]. We and others have previously reported that GRB7 is frequently overexpressed and promotes cell proliferation, cell migration and cell invasion of human cancers [10], [12], [42]. Given to its important roles as signal transduction molecules in activating oncogenic signaling pathways, numerous studies have attempted to develop inhibitors targeting to the SH2 domain of GRB7 in order to inhibiting aberrant activation of related signaling activities and eliminating cancer cells [43], [44], [45], [46]. For examples, the combination of Grb7 cyclic peptide, G7-18NATE, with chemo-drugs were capable of inhibiting breast cancer cell growth [47], [48], or with the specific peptide ligand to suppress GRB7-mediated metastasis in pancreatic carcinoma etc [11], [49]. However, the specificity and affinity are the main hurdles encountered in the development of these inhibitors as GRB7-targeted molecular therapeutics [26], [49], [50]. Here, our data show that GRB7 regulates ERK activity and the FOXM1 expression orderly and is in agreement with our previous reports [12], [21], indicating that the overexpressed GRB7 enhances ovarian cancer cell growth and cell migration/invasion through elevating ERK and FOXM1 activities. Therefore, we proposed that suppressing the aberrant activated ERK and FOXM1 should exert similar effects of using GRB7 inhibitor in inhibiting the above tumorigenic properties of ovarian cancer cells. Indeed, our *in vitro* and *in vivo* tumorigenic studies clearly show that the ovarian cancer cell growth and cell migration/invasion are remarkably reduced upon treatments of PD98059 or U01260, and Thiostrepton via targeting MEK/ERK and FOXM1 activities respectively. More importantly, the tumor suppressive effects derived from either U0126/PD98059 or Thiostrepton are equivalent. These findings seem to provide an alternative approach when developing GRB7-targeted therapeutics in ovarian cancer.

In summary, this study shows that aberrant activation of GRB7/ERK/FOXM1 signaling cascade is significantly correlated with the development of ovarian cancer. Targeting this signaling axis by MEK/ERK or FOXM1 inhibitors could obtain promising effect on the signal transduction-based therapy for this disease.

## Supporting Information

Figure S1 **Immunohistochemical analysis showed increased expressions of GRB7, ERK phosphorylation and FOXM1 were associated with advanced stage ovarian cancers.**
(TIF)

Figure S2 **Enforced expression of GRB7 increases tumor growth in mouse xenograft model.** The GRB7 stably expressing A2780cp cells (Acp-GRB7) and empty vector control A2780cp cells (Acp-V) were subcutaneously injected into the right flank of nude mice (5 mice per group). The tumor size was monitored for every 3-day. The representative pictures and bar charts show the average tumor weight of each group taken on day 18. (*P = 0.02, Student *t*-test).
(TIF)
