# Correction: Targeting GRB7/ERK/FOXM1 Signaling Pathway Impairs Aggressiveness of Ovarian Cancer Cells

**DOI:** 10.1371/journal.pone.0110304

**Published:** 2014-10-06

**Authors:** The PLOS ONE Staff

There is an error in in Figure 3. Incorrect pictures were erroneously used in Figure 3A. Please see the corrected Figure 3 here.

**Figure 3:**
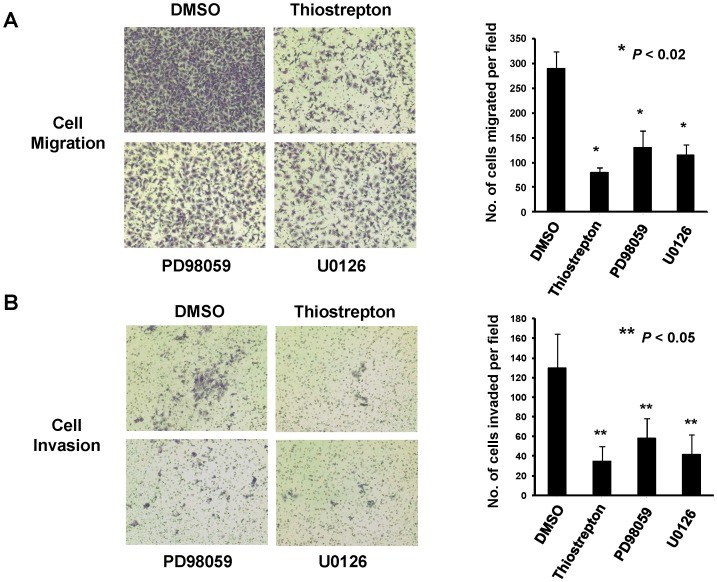
Inhibition of ERK phosphorylation or FOXM1 significantly decreased both migration and invasion of GRB7-overexpressing ovarian cancer cells. OVCA433 cells with stable expression of GFP/GRB7 (OVCA433-GRB7) were treated with DMSO as control, Thiostrepton (20 µM), PD98059 (20 µM) and U0126 (10 µM) for 6 hours and were analyzed by (A) Transwell cell migration assay. The representative pictures and bar chart showed significant reduction in the number of migratory cells through Matrigel-coated membrane in OVCA433-GRB7 cells treated with Thiostrepton, PD98059 and U0126 than DMSO control (*P<0.02, Student t-test) at 8-hour; (B) Transwell cell invasion assay. The representative pictures and bar chart showed significant reduction in the invasion rate in OVCA433-GRB7 cells treated with Thiostrepton, PD98059 and U0126 when compared with DMSO control (*P<0.05, Student t-test) at 15-hour.
