# Supplementary figures and images for: Targeting GRB7/ERK/FOXM1 Signaling Pathway Impairs Aggressiveness of Ovarian Cancer Cells
Source: PLoS One. 2012 Dec 20;7(12):e52578. doi: 10.1371/journal.pone.0052578 (PMC3527599; doi:10.1371/journal.pone.0052578)

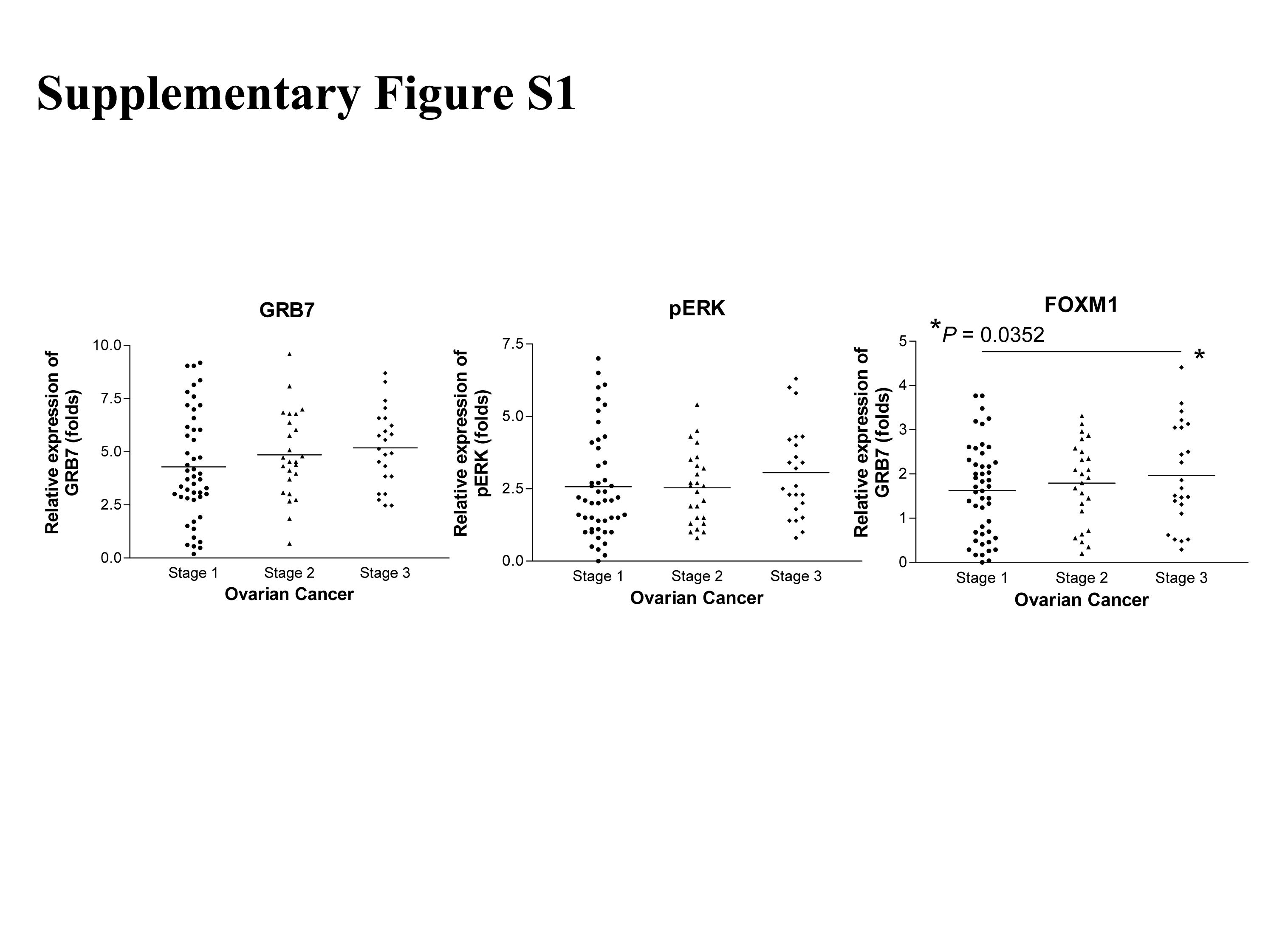

Supplement: Figure S1 — Immunohistochemical analysis showed increased expressions of GRB7, ERK phosphorylation and FOXM1 were associated with advanced stage ovarian cancers. (TIF) [file pone.0052578.s001.tif]

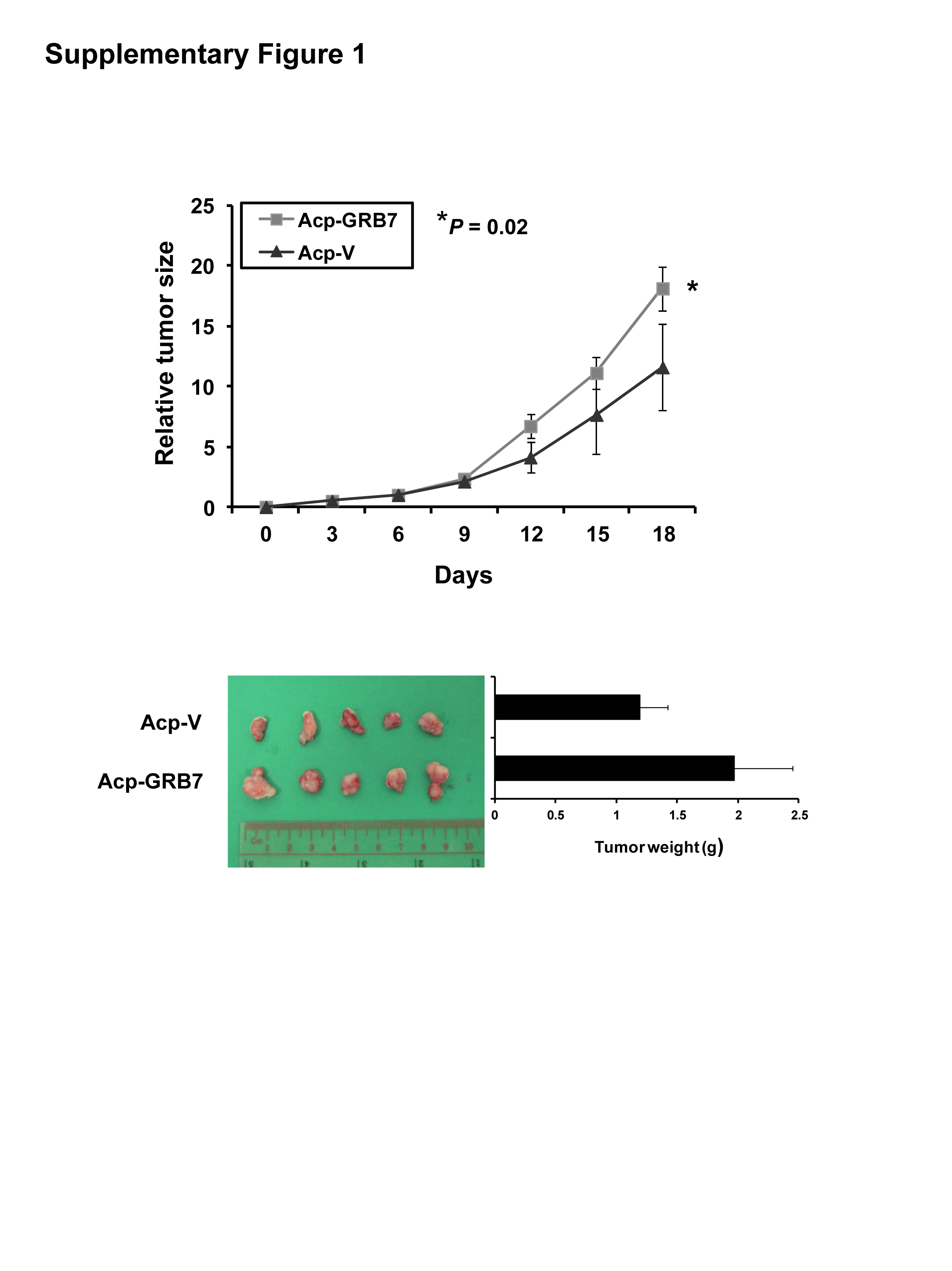

Supplement: Figure S2 — Enforced expression of GRB7 increases tumor growth in mouse xenograft model. The GRB7 stably expressing A2780cp cells (Acp-GRB7) and empty vector control A2780cp cells (Acp-V) were subcutaneously injected into the right flank of nude mice (5 mice per group). The tumor size was monitored for every 3-day. The representative pictures and bar charts show the average tumor weight of each group taken on day 18. (*P = 0.02, Student t-test). (TIF) [file pone.0052578.s002.tif]
